# Closure of Universities Due to Coronavirus Disease 2019 (COVID-19): Impact on Education and Mental Health of Students and Academic Staff

**DOI:** 10.7759/cureus.7541

**Published:** 2020-04-04

**Authors:** Pradeep Sahu

**Affiliations:** 1 Medical Education and Simulation, Centre for Medical Sciences Education, The University of the West Indies, St. Augustine, TTO

**Keywords:** coronavirus, covid-19, outbreak, pandemic, education, mental health, universities

## Abstract

The novel coronavirus disease 2019 (COVID-19), originated in Wuhan city of China, has spread rapidly around the world, sending billions of people into lockdown. The World Health Organization (WHO) declared the coronavirus epidemic a pandemic. In light of rising concern about the current COVID-19 pandemic, a growing number of universities across the world have either postponed or canceled all campus events such as workshops, conferences, sports, and other activities. Universities are taking intensive measures to prevent and protect all students and staff members from the highly infectious disease. Faculty members are already in the process of transitioning to online teaching platforms. In this review, the author will highlight the potential impact of the terrible COVID-19 outbreak on the education and mental health of students and academic staff.

## Introduction and background

The novel coronavirus disease 2019 (COVID-19) emerged at the end of December 2019 in Wuhan city of China [1]. The initial outbreak of COVID-19 in Wuhan spread rapidly, affecting other parts of China. The authorities in Wuhan took unprecedented steps and locked down the city on January 23, 2020, to lower the risk of further disease transmission [2]. Later on, the same measures were taken in other places in China. Within a few weeks, cases of COVID-19 were detected in several other countries and soon, it became a global threat [3]. The World Health Organization (WHO) declared the coronavirus epidemic a pandemic [4]. As of March 29, the virus had spread to more than 177 countries and infected more than 722,435 patients, resulting in more than 33,997 deaths [5]. Regions severely affected by major outbreaks include China, Europe, Iran, South Korea, and the United States [6]. On March 13, the WHO stated that Europe had become the new epicenter of the pandemic [7]. China took aggressive action and succeeded in reducing new cases. Unfortunately, this reduction was not the case in other parts of the world, including Iran, Italy, the US, and other European countries [8].

To respond to COVID-19, many countries have now introduced travel restrictions (both inward and outward) with the intention to prevent the spread of the epidemic. Public health experts and government officials are taking several measures, including social distancing, self-isolation, or quarantine; strengthening health facilities to control the disease; and asking people to work at home [9]. Several countries have announced the closure of gyms, museums, movie theaters, swimming pools, and places with large gatherings, inclusive of educational institutions, to fight this invisible enemy. Preliminary evidence indicated that only elderly people were getting affected and children are less susceptible to the virus. However, doctors reported a few cases of virus among children [10]. The virus should be kept away from the pediatric population because it is difficult to stop a sick child to play with friends and siblings and to hug his/her mother. Efforts to reduce the spread of the COVID-19 virus among the younger and adult populations has prompted the widespread closure of schools, colleges, universities, and other educational institutions in many countries. As of March 25, 150 countries have closed schools and educational institutions nationwide, impacting over 80% of the world’s student population. Several countries have implemented localized school closures and those closures are expected to be nationwide [11].

A large body of literature exists on the closure of educational institutions to reduce the spread of infectious disease in the community by breaking important chains of transmission [12-14]. In light of rising concern about the current COVID-19 pandemic, a growing number of universities across the world have either postponed or canceled all campus events such as workshops, conferences, sports (both intra and inter universities), and other activities. Universities have moved rapidly to transition various courses and programs from face-to-face to online delivery mode [15-16]. In this review, the author will highlight the potential impact of the terrible COVID-19 outbreak on the education and mental health of university students.

## Review

Challenges

Here are the challenges universities across the world are facing due to the COVID-19 outbreak:

Shifting from Face-to-Face to Online Classes

Worldwide, many teachers and students have been excited by the move to the online delivery mode. Faculty have already begun preparing lesson plans to deliver online teaching to their students. Online teaching is not a new mode of delivery for any university. Many faculty members get training to use online learning platforms either as the only delivery mode or as an add-on to face-to-face teaching [17]. Nevertheless, there is always a chance that some faculty who are not techno-savvy will not be able to cope up with this mode [17]. The transition to online mode has raised questions for the faculty about their capability to deal with the existing technology [18]. Furthermore, computers and IT equipment at home are now in heavy demand from parents, children, and other relatives who have to work from home. Thus, working at home is going to be a difficult task for the faculty. Also, many universities do not have enough infrastructure or resources to facilitate online teaching with immediate effect [19]. What about those students who do not have access to laptops and internet facilities at home? Is it possible to teach practicals and labs, music and art courses online? What will happen to those students whose courses cannot be taught online? The quality of online education is a critical issue that needs proper attention.

Assessment and Evaluation

Several universities have already suspended the semester-end final examinations, whereas continuous assessment will go on along with the online classes. The transition from face-to-face teaching to online delivery has a serious impact on assessments and evaluation. Although technology has been used earlier to support teaching and learning, the assessment aspect is often under-developed [20]. Applying assessments online on those courses designed for face-to-face learning is a challenging task. Students, as well as faculty, are uncertain about the procedure for administrating outstanding assignments, projects, and other continuous assessments [21-22]. Faculty members have to change the assessment types to fit online mode. It is difficult to monitor how they are taking it online and to ensure that students are not cheating during online tests [23]. Again lab tests, practicals, and performance tests are not possible to conduct online. In addition, students who do not have an Internet facility will suffer a clear disadvantage while participating in the evaluation process, which would adversely affect their grade point averages (GPAs) [24].

International Students

There are many international students studying in universities for whom travel to their home is not possible in this critical situation. While universities are closing campuses, it is important to consider that many students do not have any other accommodation facilities outside those campuses [25]. It has become a great challenge for administrators to ensure food, accommodation, and safety service for those non-national students. Students also need proper advice to protect themselves from any person-to-person contact and live in self-isolation until the situation becomes normal. Extension of stay due to the delay of examinations may cause a monetary problem. Those who manage to go home are concerned that their studies will be interrupted. At home, many students may not have the correct setup such as books, computers, and high-speed Internet connection. Again, the disruption due to COVID-19 may affect the admissions of international students for the coming academic session [26].

Travel Restrictions

The COVID-19 outbreak has created worldwide chaos for airlines. Nations across the globe are closing international borders to mitigate the outbreak [27]. University administrations are advising their staff members to postpone, until there is a return to normalcy, participation in any event that would require them to travel overseas [28-29]. It is obvious that many staff members have already paid conference registration fees and air tickets from study and travel or any other university funds. It gives rise to a state of confusion among the staff while dealing with such situations. Universities across the world are asking international students not to travel overseas and continue their studies from hostels. Students traveling overseas are putting themselves at risk of getting infected.

Mental Health

The COVID-19 outbreak has disrupted the lives of many people across the world. The worldwide rapid increase of infected cases has created a sense of uncertainty and anxiety about what is going to happen. It has also caused a tremendous level of stress among the university fraternity, inclusive of students. This stress may lead to unfavorable effects on the learning and psychological health of students [30-31]. International students staying far from home are not only worried about their health, safety, and education but they also have a huge number of concerns for the wellbeing of their families [32]. Questions arise: Are universities taking proactive measures to support the mental health and well-being of students? Do universities have professionally trained counselors who can understand such students? [25]. Students who managed to go home are worried about being unable to return to their respective institutions for further studies.

The COVID-19 pandemic may have a serious impact on the careers of this years' university graduates. They are experiencing major interruptions in teaching and assessment in the final part of their studies. They may likely graduate late due to the postponement of the final examination. Further, the graduates are going to face the severe challenges of the global recession caused by the COVID-19 crisis.

Support Services from the Universities

Universities should establish a task force to plan and deal with the crisis that is driven by COVID-19. The task force should include members from different areas within the university such as academics, human resources, facility management, health units, student affairs, enrolment services, and other relevant members. The task force should frequently meet with various subcommittees formed for the outbreak and make informed decisions as the situation evolves.

Due to the rapid increase of COVID-19 cases worldwide, universities should cancel or postpone all events, sports, workshops, conferences, and other activities for an indefinite period of time. They should avoid or reschedule meetings involving large numbers of staff or students unless it is urgent. Instead of physical meetings, they should try to conduct Zoom meetings.

It is the right time for faculty, students, and administrators to learn from this critical situation and to overcome these challenges. Online learning could be a greater opportunity as a result of this crisis. Students are young and energetic, and they are capable of learning through the online platform. Faculty can motivate the younger minds and draw them into active participation. University authorities should encourage students and faculty to stay connected through the online or any social media platform and move forward together during this extremely difficult time. Students should be provided with course instruction and other services in an online format to support academic continuity [15]. The training program should be organized as quickly as possible for the faculty members to tackle the online learning platform [17]. This force experimentation will guide universities around the world to upgrade their technical infrastructure and make online a core aspect of teaching and learning.

Students are concerned amid widespread fears that the outbreak will adversely affect their exam performance. Clear directions should be given to them regarding the procedures for administrating mid-term exams, assignments, and projects [17]. Faculty members, with the respective heads, should frame a flexible assessment guideline to keep in mind that students are not at a disadvantage [26]. If any student is not able to attend a course online due to illness or any disturbance, universities should remain as flexible as possible to ensure that he or she will not get any negative impacts in terms of grading. Some courses, such as labs, fine arts, clerkship, dance, art, and music, cannot be taught online. In such cases, the faculty can simply grade students on the work they have already done or suspend classes until things become normal.

As we witness the outbreak unfolding globally, the safety and well-being of students and staff members should be the highest priority. Universities should place an emphasis on mental health support by updating the health guidelines and providing online guidance and lectures to offer strategies for managing stress when coping with the pandemic. Any student experiencing feelings of heightened anxiety about COVID-19 should be provided with proper psychological support well in time [30]. Further, universities should pay more attention and systematic support to vulnerable international students [25]. Hostels and residences should remain open for students who are unable to return to their homes. Universities should consider matters relating to financial support and the general living expenses of needy students. To overcome the challenges of the global recession, graduates should be encouraged to stay in school and pursue another degree. Universities, governments, banks, and student loan companies should support graduates in various ways, including direct case support and temporary suspension of students’ loan payment.

Along with the existing challenges in managing teaching and evaluation during the COVID-19 outbreak, universities have to prepare a road map to accommodate admissions for coming academic sessions. Admission officials should accept applications from prospective students online and offer them more flexible admissions processes [25-26]. Revised information should be updated on the university website.

## Conclusions

In the emerging and ever-changing COVID-19 context, universities should implement a number of measures to slow the spread of the virus. Students and staff should receive regular information through emails and university intranets. The health and safety of students and staff should be the top priority. Proper counseling services should be available to support the mental health and well-being of students. Authorities should take the responsibility of ensuring food and accommodation for international students. Faculty members should embrace technology and pay careful attention to student experiences to make the learning rich and effective.
